# Unraveling Whole-Genome Sequence and Functional Characterization of *P. megaterium* PH3

**DOI:** 10.3390/foods13223555

**Published:** 2024-11-07

**Authors:** Xiaohan Zhang, Junbo Liang, Dong Zhang, Liang Wang, Shuhong Ye

**Affiliations:** 1 School of Food Science and Technology, Dalian Polytechnic University, Dalian 116034, China; 20173083200043@xy.dlpu.edu.cn (X.Z.); 221720860001048@xy.dlpu.edu.cn (J.L.); 211720860000992@xy.dlpu.edu.cn (D.Z.); wangliang@dlpu.edu.cn (L.W.); 2 State Key Laboratory of Marine Food Processing and Safety Control, Dalian 116034, China

**Keywords:** *Priestia megaterium*, whole genome, safety evaluation, toxicity evaluation, biological characteristics

## Abstract

*Priestia megaterium* (*P. megaterium* PH3) is an endophytic bacterium isolated from peanuts. It has natural resveratrol production ability and shows potential application value. This study analyzed its genetic function and metabolic mechanism through whole-genome sequencing and found that the genome size is 5,960,365 bp, the GC content is 37.62%, and 6132 genes are annotated. Functional analysis showed that this strain contained 149 carbohydrate active enzyme genes, 7 secondary metabolite synthesis gene clusters, 509 virulence genes, and 273 drug-resistance genes. At the same time, this strain has the ability to regulate salt stress, low temperature, and hypoxia. Genomic analysis reveals a stilbene-synthase-containing type III polyketide synthase gene cluster that contributes to resveratrol synthesis. A safety assessment showed that the strain is non-hemolytic, does not produce amino acid decarboxylase, and is not resistant to multiple antibiotics. In the mouse model, *P. megaterium* PH3 did not have significant effects on body weight, behavior, or physiological indicators. These results provide important basic data and theoretical support for its industrial application and the research and development of plant protection agents.

## 1. Introduction

*Priestia megaterium* (*P. megaterium*) is a multifunctional microorganism with broad application prospects in industrial production, agriculture, and environmental protection [1]. In industrial production, *P. megaterium* can be used to produce a variety of enzymes and biological products, which not only enhances its application potential in the fields of biopharmaceuticals and functional food [2] but also makes it an ideal choice for the human food industry and livestock feed additives due to its stability and non-pathogenicity [3]. With the rapid development of microbial genomics, comprehensively analyzing the genome information of microorganisms has become an important way to understand their biological characteristics, metabolic ability, and potential applications [1]. In recent years, the development of genomics has solved the genetic diversity and strong environmental adaptability of *P. megaterium* [4]. The discovery of many important genes, such as gas vesicle protein and P450 cytochrome monooxygenase, provides a new basis for its biotechnological applications [2]. Genomic analysis of Bacillus megaterium isolated from oilseed rape roots reveals clusters of genes such as bacteriocin, polyketide synthase (PKS), and non-ribosomal peptide synthase (NRPS). These clusters are associated with antimicrobial compounds like sulphate, bacillus, and bacillusin, indicating the potential for inhibiting plant pathogens [5]. Genomic analyses of different strains of Priestia megaterium show that they generally contain genes for synthesizing polygamma-glutamic acid pods similar to those of *Bacillus anthracis*. The main differences between strains lie in the number of plasmids and giant plasmids, as well as the genes they carry. Clinical strains tend to be more abundant in these aspects compared to model strains, reflecting their greater adaptability to the medical environment [6]. In addition, the genetic operation of the bacteria is relatively easy, and a variety of genetic tools and mutants have been developed to support research and production needs [4].

It is of great scientific and practical significance to evaluate the safety of endophytes that produce natural products. Endophytes are regarded as an important source of natural products, and their safety assessment is crucial to ensuring medicinal potential [3]. In addition, the impact of endophytic bacteria on host plants may affect the stability of the ecosystem, so understanding their safety helps to maintain ecological balance [7]. In recent years, with the increasing demand for environmentally friendly biological protection agents in agriculture and biotechnology, especially the application of some endogenous bacteria, it has become particularly important to study the function and safety of these microorganisms [8]. In biomedical research, evaluating the safety of microorganisms is a key step to ensure that they do not cause adverse reactions in clinical applications. As part of biological agents, the safety assessment of bacteria is particularly important because they may directly or indirectly affect human health [9]. The mouse model is often used as an experimental group to evaluate the safety of bacteria because of its physiological and genetic similarity with humans. The possible effects of bacteria in the human body are simulated through mouse models so as to preliminarily predict their potential impact on the human body [10].

Through whole-genome-sequencing analysis, this study deeply studies the metabolic system, pathogenic mechanism, intracellular regulation, and functional protein of the *P. megaterium* PH3 and comprehensively analyzes the biological characteristics of the strain. In addition, the interaction between pathogenic microorganisms and hosts is also explored to understand their ecological role in habitats and their potential application value. Additionally, the safety of the strain, including drug-sensitivity experiments, hemolysis properties, metabolic ability and bioamine research, and drug susceptibility tests, is studied to confirm its feasibility and safety in application. Finally, the influence of bacteria on the physiological and pathological state of mice is evaluated through the gastric perfusion toxicity test of mice, which provides a scientific basis for the potential application of the strain and further clinical application.

## 2. Materials and Methods

### 2.1. Strains and Culture Conditions

The strain *P. megaterium* PH3 is stored in China’s typical culture preservation center. The storage address is No. 299, Bayi Road, Wuchang District, Wuhan City, Hubei Province. The storage number is CCTCC NO: M 20222035. After the strain is treated, the strain *P. megaterium* PH3 stored at −80 °C is revived. It is then inoculated on the yeast extract peptone dextrose medium culture medium for activation, and the 4 °C preservation is carried out for subsequent experiments.

### 2.2. Kirby–Bauer Disk Diffusion Tests

The Kirby–Bauer (K–B) disk diffusion method is an important experimental technique used to determine the sensitivity of bacteria to antibiotics. Through the diffusion of antibacterial drugs on the culture medium, observe whether there is a growth inhibition zone and infer whether the growth of bacteria is inhibited [11]. For specific experimental operations, refer to the methods reported in the previous research and make slight modifications [12]. After the target strain is activated, it is coated on the most suitable culture medium for growth, and a piece of paper containing quantitative antibacterial drugs is applied to the solid culture medium inoculated with bacteria to be tested. It is cultured at 28 °C for 20 to 24 h to determine the growth inhibition zone. Resistance assay standards refer to the Clinical and Laboratory Standards Institute [13]. Three groups of parallel experiments are set up for each experiment to ensure the reliability of the results.

### 2.3. DNA Extraction and Whole-Genome Sequencing

Genome sequencing was performed using *P. megaterium* PH3 cultured for 24 h. The brief description is that the cultured strain was centrifuged at 10,000 rpm/min for 10 min at 4 °C to isolate the culture solution to obtain the strain, and the bacterium was washed with phosphate buffered solvent until the wash solution was colorless, and the culture solution was completely removed, and then it was set aside for subsequent sequencing.

Sequencing was performed by Shanghai Meiji Biomedical Technology Co., Ltd. (Shanghai, China), in which the Wizard^®^ Genome DNA Purification Kit (Promega, Madison, WI, USA) is used for the extraction of genomic DNA. The purity of DNA is detected by NanoDrop 2000, the concentration is determined by Quantum Fluorometer, and the integrity of DNA is analyzed by agarose gel electrophoresis. The subsequent sequencing work was carried out on the second-generation sequencing platform Illumina Hiseq × 10, using the PE150 (paired-end) sequencing method. The final sequencing data are combined with the second-generation and third-generation sequencing technology, that is, the test results of the Illumina Hiseq and PacBio platforms.

### 2.4. Genome Assembly and Optimization

Genomic reads were obtained by CASAVA-based identification of sequencing image signals using the Illumina platform. For the obtained data, firstly, Fastp 0.20.0 was applied to cut the low-quality data in order to obtain clean data, and, secondly, SOAPdenovo2 and Meryl 1.3 were used to assess the data quality of GC content, genome repeatability, size, and plasmid, and the pure data that met the criteria were filtered out to be presented in GC depth distribution plots and K-mer frequency distribution plots. Again, the initial assembly of the genome was performed using the short sequence assembly software SOAP denovo 2 [14] to form scaffolds. The final construction of the bacterial genome was performed using Unicycler v0.4.8 [15] software for three-generation sequence assembly and combined with Pilon v1.22 software for sequence correction.

### 2.5. Genome Assembly Genome Prediction

For the coding sequence in the genome, we use Glimmer 2 to predict the chromosomal genome [16] and use GeneMarkS 4.3 to analyze the plasmid genome [17] to obtain the nucleic acid sequence and amino acid sequence of the functional gene. In addition, tRNAscan-SE v2.0 software is used to predict tRNA [18], Barrnap 0.9 is used to predict rRNA, and BLAST 2.3.0 is used to verify core genes. For the prediction of serial repeat sequences, we use Tandem Repeats Finder 4.09.1 to analyze [19] and, at the same time, identify and classify sequences similar to known repeat sequences through RepeatMasker 4.1.4. Finally, Single Molecule Real-Time (SMRT) analysis software, which is a three-generation sequencing technology developed by Pacific Biosciences that enables the determination of DNA sequences by monitoring the synthesis of each base by DNA polymerase in real time, is used to discuss the methylation modification of DNA.

### 2.6. Annotation Strategy and Database Utilization

Detailed functional information was obtained by comparing the gene sequences with several authoritative databases. The databases specifically used include a widely covered Non-Redundant Protein (NR) database, a Swiss-Prot database known for its high reliability, a Pfam database that provides genetic domain information, a Clusters of Orthologous Groups of proteins (COG) database that focuses on gene family functional annotations, a Gene Ontology (GO) database that standardizes biological terms, and the Kyoto Encyclopedia of Genes and Genomes (KEGG) database that analyzes genetic functions. Through comparative analysis with the above databases, we performed systematic annotation based on sequence homology, structural domains, and functional classification of genes to identify key enzymes and metabolic pathways, laying the foundation for functional identification of genes.

### 2.7. Analysis of Metabolic System

Use the carbohydrate active enzymes (CAZy) database to explore the functional diversity of CAZy in strains, especially the role of degradation, modification, and formation of glycoside bonds. The database provides a comprehensive classification system for identifying and classifying enzymes involved in carbohydrate metabolism. These enzymes include glycoside hydrolase, glycotransferase, polysaccharide lysase, carbohydrate esterase, carbohydrate-binding module, and auxiliary oxidoreductase. Through systematic analysis, the comprehensive function of CAZy in the strain in carbohydrate metabolism is revealed.

In the field of biology, the biosynthesis process of secondary metabolites is usually regulated by a series of genes, and the proteins encoded by these genes have the function of complex enzymes. As a bioinformatics tool, the AntiSMASH database can efficiently identify and annotate gene clusters responsible for the synthesis of secondary metabolites. Through the query and functional prediction of the gene cluster system of potential natural product synthesis in the bacterial genome, the recognition and understanding of the biosynthesis pathway of secondary metabolites of the strain are enhanced.

### 2.8. Analysis of Pathogenicity

Adopt a variety of databases and software tools, including VFDB 20230407, CARD v3.2.6, PHI 4.9, ResFinder 4.3.1, KEGG 202209, TCDB v20200917, Pfam 33.1 and Diamond 0.8.35, ResFinder_v4.1.0, signalP 4.1, and Tm. Hmm 2, hmm Er3, etc., to analyze the virulent gene, drug-resistance gene, pathogen–host interaction, drug-resistance gene Resfinder, secretory system, secretory protein, transporter protein, transmembrane protein, and two-component regulatory system of bacteria. Through these methods, the pathogenic mechanism of bacteria is comprehensively analyzed, including the expression of toxic factors, the generation of drug resistance, the interaction with the host, and the key secretion and transport systems. These analyses provide important molecular-level information for understanding the pathogenicity and adaptability of bacteria.

### 2.9. Safety Evaluation

#### 2.9.1. Experimental Analysis of Hemolysis

Analyze whether the strain has hemolytic properties, select Colombian blood agar for evaluation, and operate according to the previously reported method [19]. The activated strains were inoculated into Colombian blood agar containing 5 g/100 mL of human blood by lineation and cultured for 48 h at 28 °C. The hemolysis type of the strain is judged by observing the hemolysis phenomenon around the colony. If there is a transparent hemolytic ring around the colony, it is determined to be β hemolysis; if the hemolytic ring is green, it is determined to be α hemolysis; if no hemolytic ring is formed, it is determined to be γ hemolysis, that is, no hemolytic phenomenon. In addition, Listeria monocytogenes were used as a positive control in the experiment to ensure the accuracy and comparability of the experimental results.

#### 2.9.2. Experimental Analysis of Indigo Matrix

The indigo matrix test aims to evaluate whether bacteria can decompose tryptophan in proteins to produce indole based on the principle of the color reaction of indole and dimethylaminobenzaldehyde to form a red compound [20]. The strain to be tested was inoculated into peptone water medium at 28 °C and incubated for 48 h. After that, 8 to 10 drops of dimethylaminobenzaldehyde reagent were added to the culture solution, and the subsequent color change was carefully observed. *E. coli* ATCC 25922 served as a positive control to validate the efficacy of the process and reagents. Meanwhile, a non-inoculated protein broth medium acted as a control to eliminate the influence of non-biological factors on the outcomes [21].

#### 2.9.3. Amino Acid Decarboxylase Experiment

Identify the ability of experimental strains to produce specific biological amines during the culture process. The strains were transferred to a decarboxylase detection medium containing 0.1% of specific amino acids (e.g., tyrosine, histidine, lysine, or guanine) at 28 °C and cultured for 72 h. The strain has amino acid decarboxylase activity, which can decompose amino acids to produce alkali; the pH rises, and the color changes from yellow to purple, the result is positive, indicating that specific biogenic amines can be produced. If the color of the culture solution remains yellow, the result is negative. In addition, this experiment also uses *E. coli* ATCC 25922 as a positive control to verify the accuracy of the experimental method [22].

### 2.10. Experimental Analysis of Toxicology

#### 2.10.1. Animal Experimental Design

The experiment selected 6–8-week-old SPF-grade male Balb/C mice, weighing about 20 ± 2 g, purchased from Liaoning Changsheng Biotechnology Co., Ltd. (Benxi, China). All animal experiments strictly follow the guiding principles of the Ethics Committee of Experimental Animals of Dalian University of Technology (SYXK2017-0005). The mice adapt to the environment for a week before the experiment. The light cycle alternates day and night, the humidity is maintained at 50 ± 5%, the temperature is controlled at 23 ± 2 °C, and all animals can freely drink SPF-grade drinking water and feed. In the experiment, a total of 40 mice were selected and randomly divided into three groups (8 in each group): control group (sterile physiological saline, Control), low-dose group (1.5 × 10^5^ CFU/mL, LC), and high-dose group (1.5 × 10^10^ CFU/mL, HC) [23].

All treatment groups use gastric lagation, each group once a day; each gastric irrigation volume is 0.5 mL, and it is treated continuously for 4 weeks. During the treatment period, the weight of the mouse was recorded every day. The mouse was fasted for 12 h the day before treatment, and then blood was taken through the eyeball and the mouse was executed. All animal handling was carried out in accordance with Directive 2010/63/EU of the European Parliament and of the Council of the European Union on the protection of animals used for scientific purposes (22 September 2010). The sample taken was soaked in 4% polyformaldehyde for slicing experiments, while the biochemical analysis sample was stored at −80 °C for subsequent experimental use [24,25].

#### 2.10.2. Weight Measurement and Behavior Observation

In order to comprehensively evaluate the health status of mice, weight measurement was selected at the same time every day (9 a.m.) during the experiment, and the data obtained was recorded. In order to further understand the effect of the drug on the behavior of mice, the activity, social interaction, eating and drinking behaviors of the mice should be carefully observed within 4 h after the drug is given. In addition, any abnormal behavior of mice, such as muscle twitching, tremor, abnormal gait, aggressive behavior, or withdrawal of social behavior, is recorded and described in detail.

#### 2.10.3. Determination of Visceral Weight Coefficient

For the heart, liver, spleen, lungs, kidneys, and other major internal organs of mice, wash the organs with physiological saline to remove blood and residual tissues. After cleaning, use filter paper to absorb the moisture on the surface of the organs and measure the moisture weight of each major internal organ with an electronic balance. The weight coefficient of each internal organ was calculated with the formula as follows: organ weight coefficient = (organ wet weight/mouse weight) × 100%. This indicator is used to quantitatively analyze the relationship between organ weight and the overall weight of mice, so as to evaluate the relative size of organs and potential pathological changes.

#### 2.10.4. Pathological Section Analysis

For preparation of pathological sections, the tissue sample is first fixed in a 4% polyformaldehyde solution to maintain the integrity of its morphological structure. Then, the moisture in the tissue is gradually removed through the gradient dehydration step of ethanol. Xylene is used as a transparent agent to replace the moisture and lipids in the tissue. The transparency process should be strictly controlled within 1.5 h to prevent damage to the organizational structure. After that, the tissue sample was buried in paraffin and prepared into a 5 micron-thick slice using a fully automatic slicer. The slice is stained with Sumujing–Yihong (H&E) and finally sealed with neutral gum. Images were recorded using a panoramic digital slice scanner for further analysis [26].

#### 2.10.5. Analysis of Serum Oxidative Stress

The antioxidant indices in serum samples were determined using the Glutathione Peroxidase Assay Kit, Total Superoxide Dismutase Assay Kit, and Malondialdehyde Assay Kit (Nanjing Jianjian Institute of Bioengineering, Nanjing, China), which mainly consisted of enzyme activities, including superoxide dismutase (SOD) and glutathione (GSH), and malondialdehyde (MDA) content levels. The measurement method refers to the manufacturer’s instructions.

### 2.11. Statistical Analysis

All data were presented as mean ± standard deviation, and the graphs were processed by Originpro 8.5. Multiple comparative analysis was performed using Duncan’s test in SPSS 17.0 (Statistical Package for the Social Sciences Software, SPSS Inc., Chicago, IL, USA). The significance level was set as *p* < 0.05, marked with different number of asterisks to indicate the degree.

## 3. Results and Discussion

### 3.1. P. megaterium PH3 Whole-Genome Analysis

In recent years, resveratrol has attracted much attention for its remarkable antioxidant and anti-inflammatory properties. In order to deeply explore the endogenous bacteria that produce resveratrol and their potential application value, we conducted a whole-genome-sequencing study. By analyzing its metabolic system, pathogenic mechanism, intracellular regulation, and functional protein, as well as the interaction between pathogenic microorganisms and the host, it aims to reveal the biological characteristics of the bacteria.

#### 3.1.1. Genome Evaluation

**Figure 1:**
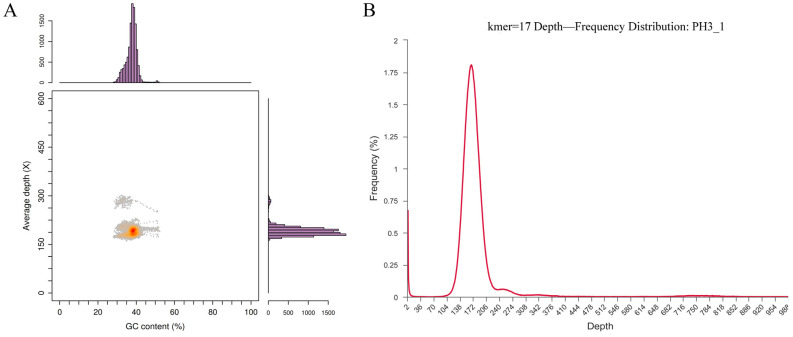
Genomic evaluation. (**A**) GC depth distribution analysis (Depth of staining indicates enrichment); (**B**) K-mer frequency distribution analysis.

In order to ensure the accuracy of the genome, this study evaluates the quality of the genome sequences initially assembled. Different types of microorganisms (such as bacteria, fungi, etc.) have relatively stable GC contents [27]. The genome GC content of different microorganisms usually has a specific range. If the GC content of the genome obtained by sequencing is significantly out of the expected range, it may indicate that the genome is contaminated [28]. The distribution of GC depth is used to analyze whether the genome is contaminated. The distribution map of GC% content is shown in Figure 1A. The results show that the GC% content is distributed centrally, and there is no obvious GC bias; the coverage depth of the histogram on both sides shows the centralized distribution, and most of the points are concentrated in a narrow range. The above results can be obtained, and there is no pollution in the genome.

The plasmid distribution and pollution of the genome are further evaluated by using the frequency distribution of K-mer. The diversity and frequency of K-mer can initially reflect the repeatability and complexity of the genome [29]. As shown in Figure 1B, the appearance of high-frequency peaks indicates the existence of plasmids. There is no multi-peak appearance in the frequency distribution, and there is no obvious horizontal block distribution, indicating that there is only one type of DNA and no pollution risk. There is no obvious group block in the vertical direction, indicating that there is no repeated sequence of species. The above results can be obtained, and the genetic sequence is not polluted.

#### 3.1.2. Genome Loading and Prediction

**Figure 2:**
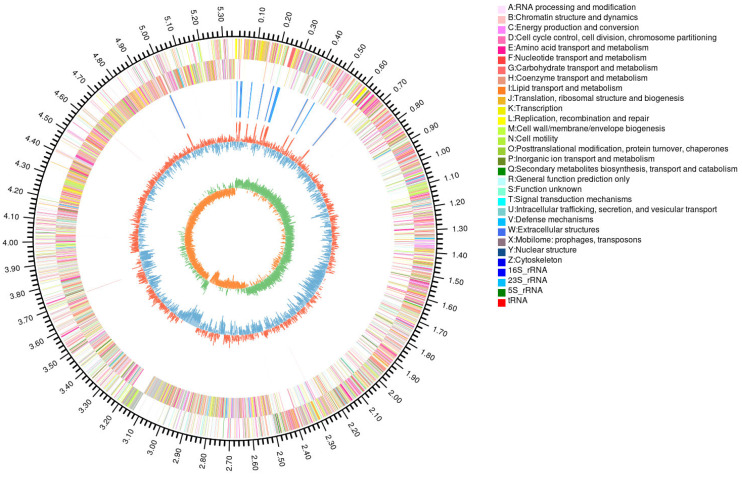
*P. megaterium* PH3 genome circle map (note: the outermost circle, genome size; the second circle, coding sequence (CDS) on the positive chain; the third circle, CDS on the negative chain; the fourth circle, rRNA and tRNA; the fifth circle, GC content; the innermost circle, GC skew value).

After using Illumina HiSeq and PacBio platforms for sequencing, the genome circle chart is shown in Figure 2. The genome assembly analysis shows that the *P. megaterium* PH3 genome sequence is assembled into eight scaffolds, with a total length of 4,318,106,144 bp; the longest is 317,278; and the N50 is 11,391 bp. The genome of this organism is composed of one chromosome and seven plasmids. The genome size is 5,960,365 bp, and the GC content is 37.62% (Table 1). As the core of the genome, chromosomes carry basic genetic information, while plasmids, as additional genetic components, carry additional genes, including resistance genes and metabolic genes [30]. These plasmids are usually related to the environmental adaptability of organisms, giving the host characteristics such as drug resistance and heavy metal tolerance. The existence of multiple plasmids may indicate that the organism has the ability to survive and reproduce in a variable environment. In addition, some plasmids may encode specific metabolic pathways so that the host can effectively utilize specific nutritional resources or decompose harmful substances [31]. The phenomenon of the gene horizontal transfer of plasmids in bacteria indicates that the organism may have significant ability to exchange genetic materials, which is crucial for rapid adaptation to environmental changes [32]. In summary, these results suggest that the organism has high genetic diversity and good environmental adaptability.

This study reveals the complexity and functionality of the genome of the organism through genome sequencing and annotation. A total of 6132 genes are annotated, covering 81.83% of the genome, indicating that most regions of the genome are involved in coding functional proteins. In addition, the genome encodes 140 tRNAs distributed at 21 sites, as well as 47 rRNAs and 115 sRNAs. These non-coding RNA molecules play a key role in gene expression regulation and protein synthesis, accounting for 0.3% of the genome in total. It is especially worth noting that four methylation sites have been found, including 2 m4C and m6A-modified bases, implying the epigenetic mechanism of gene expression regulation. The existence of pseudogenes was not predicted in the study, which may indicate the high functionality of the genome or the limitations of annotation techniques (Table 2). These results not only provide important information for understanding the molecular biological characteristics and environmental adaptability of the organism but also lay the foundation for future genetic function research, biotechnology application, and evolutionary biology research.

#### 3.1.3. Genetic Annotations

**Figure 3:**
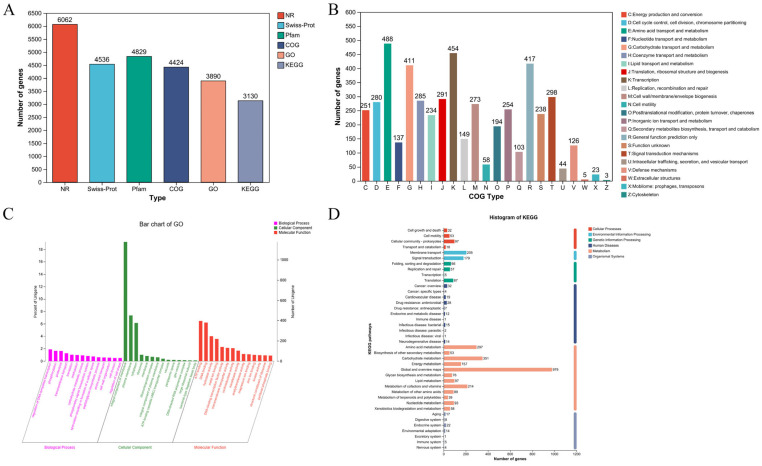
Gene annotation. (**A**) Gene base annotation analysis; (**B**) Non-Redundant Protein Database (COG) annotations; (**C**) Gene Ontology (GO) annotation; (**D**) Kyoto Encyclopedia of Genes and Genomes (KEGG) annotation.

With the continuous progress of sequencing technology and calculation methods, the importance of gene annotation in biomedical research and application is increasingly prominent [33]. Gene annotation is the core process in bioinformatics. By identifying and describing genes in genome sequences and their functions, it provides us with key information to deeply understand the structure and function of the genome of organisms [34]. This study reveals the composition and distribution of genes through multi-database annotation analysis and predicts the function of genes, mainly including NR (Non-Redundant Protein sequence information), Swiss-Prot (protein function and characteristic analysis), Pfam (protein structure and function), COG (evolutionary classification of systems and functions), GO (the role of molecular function, cell components, and biological processes), and the KEGG database (integrating information on genomes, chemicals, and biological pathways). The results show that a total of 6062 genes were annotated in the NR database: 4536 in Swiss-Prot, 4829 in Ptam, 4424 in COG, 3890 in GO, and 3130 in KEGG (Figure 3A). These databases provide a comprehensive analysis of genome data and effectively transform genome sequence information into an understanding of biological functions.

This study deeply explores the biological function and characteristics of *P. megaterium* PH3 through comprehensive genome annotation analysis. COG analysis shows that genes are mainly concentrated in several key functional classifications, including amino acid transport, carbohydrate transport and metabolism, transcription, signal transduction mechanism, etc. It shows that strains have important ecological adaptability in the acquisition and utilization of amino acids and carbohydrates (Figure 3B). GO annotation reveals the extensive participation of genes in molecular functions such as ATP binding, hydrolase activity, and DNA binding, highlighting their key role in energy metabolism, genetic regulation, and metabolism. In cell component analysis, genes are mainly located in membrane structure, plasmic membrane, and cytoplasm, indicating the characteristics of the strain in terms of structure and environmental interaction (Figure 3C). KEGG analytical strains are significantly active in the metabolic pathway, especially in the metabolism of carbohydrates and amino acids as well as in the metabolism of cofactors and vitamins. It shows that the strain can efficiently acquire nutrients and convert energy, showing its strong metabolic ability in environmental adaptability. At the same time, the significant enrichment of membrane transport and signal transduction pathways reveals that the strain may play an important role in regulating the interaction between cells and the environment (Figure 3D). In general, the genome annotation results reveal the outstanding biological characteristics of strains in metabolism, energy acquisition, signal transmission, and environmental adaptation and show their potential functions and adaptability as microorganisms in specific ecological positions.

### 3.2. Analysis of the Metabolic System of P. megaterium PH3 Genome

Genomic metabolic system analysis, a key aspect of systems biology, delves into biometabolic pathways, including carbohydrate-active enzyme annotation and secondary metabolite gene cluster analysis. This analysis enhances our understanding of organismal metabolic networks, nutrient transformation, and environmental adaptation mechanisms. It also identifies biomarkers through metabolic enzyme annotation, aiding in disease diagnosis and drug development. Moreover, studying secondary metabolite synthesis gene clusters fosters new drug and natural product discovery, offering a holistic view for systems biology and advancing our grasp of metabolic regulation mechanisms [35].

#### 3.2.1. Annotated Analysis of CAZy

**Figure 4:**
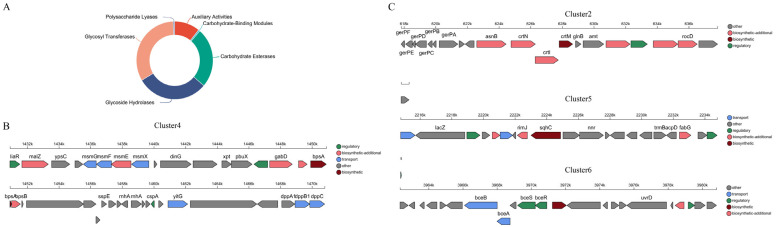
Analysis of the metabolic system of *P. megaterium* PH3 genome. (**A**) Carbohydrate-active enzymes (CAZy) functional classification map; (**B**) Type III Polyketide Synthase (T3PKS) gene cluster; (**C**) terpene gene cluster.

The CAZy database is an important bioinformatics resource, focusing on carbohydrate active enzymes [36]. The CAZy results of the genome show that there are 149 genes coding carbohydrate active enzymes, which shows that they can metabolize a variety of carbohydrates (Figure 4A). Among them, 49 genes encode glycotransferase, 45 genes encode glycoside hydrolase, and 37 genes encode carbosterase, indicating that the strain has diverse metabolic pathways in the synthesis and decomposition of complex polysaccharides and the modification of sugar molecules [37]. In addition, 16 gene-coded auxiliary active enzymes, which work together with the main hydrolytic enzymes to enhance the degradation ability of complex carbohydrates, and 1 gene-encoded carbohydrate binding module help enzymes to identify and adhere substrates, improving enzymatic efficiency [38]. One gene encodes polysaccharide lysase, which can lyse polysaccharide and produce oligomers with novel physicochemical properties, opening up a new way for the research and application of polysaccharides. These enzymes are crucial to the synthesis, metabolism, and transport of carbohydrates, affecting the survival and adaptability of strains. Metabolic analysis helps to understand their ecological role and provide important ideas for basic and applied research to further explore their biological characteristics and potential application value.

#### 3.2.2. Analysis of Secondary Metabolite Synthetic Gene Clusters (DGCs)

Genome secondary metabolite synthesis gene cluster analysis reveals that the strain has the potential to produce a variety of biologically active molecules, including lanpeptides, terpenes, phosphinate, type III polyketone synthase, and iron carriers (Figure 4B,C; Table 3). These secondary metabolites may show significant diversity in biological and ecological functions. In particular, type III polyketone synthase is closely related to the synthesis of resveratrol precursor [39,40], and stilbene synthase (STS) (astragase) is a member of type III polyketone synthase [41]. The chalcone synthase (CHS) superfamily is the main speed-limiting enzyme in the pathway to synthesize resveratrol in plants [42]. Research shows that STS in plants is highly specialized, and the synthesis of resveratrol will be affected when the substrate changes [43]. In addition, at present, only plant STS is used to produce resveratrol by microorganisms through genetic engineering transformation [44]. In the whole-genome analysis of fungi, it was found that the key enzymes that regulated the biosynthesis pathway of resveratrol were 4-coumarate:coenzyme A ligase (4CL) and CHS, while no key enzyme STS related to the synthesis of resveratrol in plants was found [45]. From this, it can be inferred that there are differences in the synthesis pathways of resveratrol in different species. The above results not only provide theoretical support for the metabolism and synthesis of resveratrol by bacteria but also provide important genetic resources for the metabolism and synthesis of resveratrol by engineering bacteria.

Therefore, this discovery may make a significant contribution to the synthesis of resveratrol by bacteria. The abundance of terpenes and their physiological functions may affect the biosynthesis efficiency of resveratrol [46]. There are also studies on regulating the rational conversion of terpenes and resveratrol in engineering bacteria to obtain the target [44]. This series of results further shows that the strain has the potential to enhance the synthesis of resveratrol in the field of metabolic engineering. Therefore, these findings not only help to deeply understand the metabolic characteristics of the strain but also provide an important research direction for future biosynthesis research and resveratrol production optimization.

### 3.3. Pathogenic System Analysis of P. megaterium PH3 Genome

The analysis of the pathogenic mechanism of bacteria can identify virulence genes and drug-resistance genes to reveal the pathogenic potential and drug resistance of bacteria [47].

#### 3.3.1. Prediction of Virulence Genes

**Figure 5:**
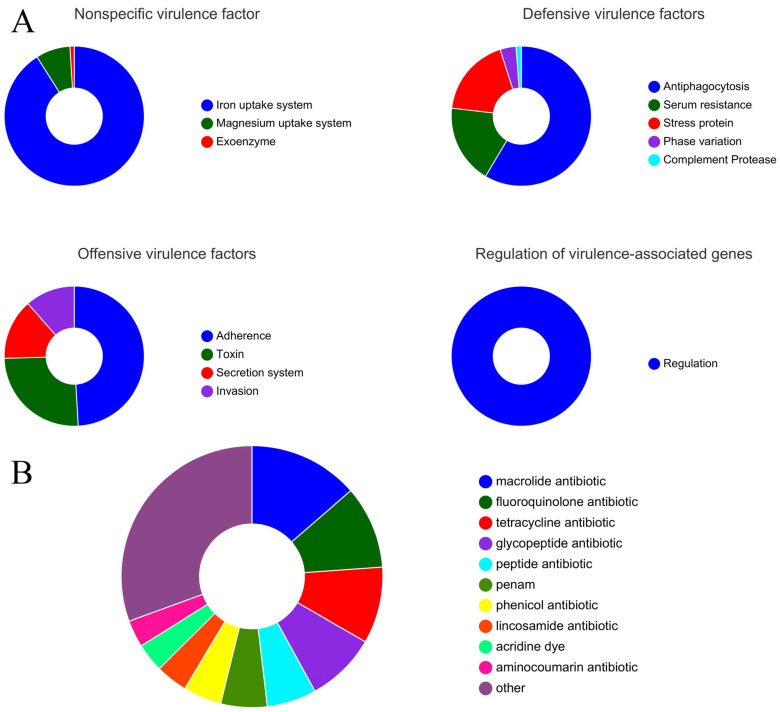
System analysis of pathogenic disease. (**A**) Virulence factor statistic; (**B**) antibiotic-resistance gene.

In this study, through in-depth bioinformatic analysis of the genome of the strain, 509 virulence genes were identified, revealing their various biological characteristics and potential application value (Figure 5A). These genes are divided into non-specific virulence factors, defensive virulence factors, offensive virulence factors, and virulence-related regulatory genes according to their functions and characteristics. The existence of non-specific virulence factors indicates that the strain can effectively obtain essential trace elements in the host, and the identification of defensive virulence factors shows that it has the ability to escape the host’s immune system. The detection of offensive virulence factors further confirms its aggressive and pathogenic potential, and the virulence-related regulatory genes may It is a key factor in adapting to changes in the host environment and regulating the expression of virulence force [48].

Among them, non-specific virulence factors include 92 iron absorption system genes, 8 magnesium absorption system genes, and 1 exogenous enzyme gene, indicating that the strain has a significant survival advantage in a nutrient-deficient environment. In terms of defensive virulence factors, the strain has 48 antiphagocytic functional genes, 15 serum drug-resistance genes, and 15 stress protein genes, which enhances its ability to resist the host’s immune system. In addition, the performance of offensive virulence factors, including 81 attachment genes, 42 toxin genes, 23 secretory system genes, and 19 invasion genes, further demonstrates its strong pathogenicity and infectious ability. Finally, the existence of 40 virulence-related regulatory genes suggests that the strain can effectively regulate the expression of virulence factors. In summary, the prediction results of this study show that the strain, as a potential pathogen, has a complex virulence gene spectrum, which gives it the ability to survive, reproduce, and pathogenically infect the host body. These findings not only provide important clues for understanding the pathogenic mechanism of the strain but also provide potential targets for the development of new prevention and treatment strategies.

#### 3.3.2. Prediction of Drug-Resistance Genes

This study predicts and analyzes the drug-resistance genes of a strain, aiming to provide scientific guidance for clinical treatment, new drug development, infection control, and public health policies [47]. The results show that a total of 273 drug-resistant genes have been identified in this strain, and the specific distribution is as follows: 69 macrocyclic antibiotics, 52 fluoroquinolones, 48 tetracyclines, 44 glycopeptides, 31 peptides, 29 penicillins, and 24 phenolquinolines (Figure 5B). These results reveal the extensive resistance of this strain to a variety of antibiotics, which may lead to a decrease in clinical treatment effects and increase the difficulty of infection control [49]. The ResFinder database (https://bitbucket.org/genomicepidemiology/resfinder/src/master/, accessed on 23 October 2024) contains experimentally verified drug-resistance genes. According to the database prediction, only one drug-resistant gene (Isa) was found, located on the plasmid (Table 4). The above results show that the strain may be drug-resistant and can transfer genes through plasmids. Therefore, an in-depth understanding of the drug-resistance characteristics of this strain is of great significance for infection monitoring and rational use of antibiotics and provides an important basis for optimizing clinical treatment plans and formulating effective public health strategies.

### 3.4. Internal Cell Regulation and Functional Protein Analysis

The two-component regulation system and transporter protein analysis of strain genes aim to comprehensively understand the signal transmission, material transport, and external interaction mechanisms of bacteria in different environments.

#### 3.4.1. Analysis of a Two-Component Regulation System

**Figure 6:**
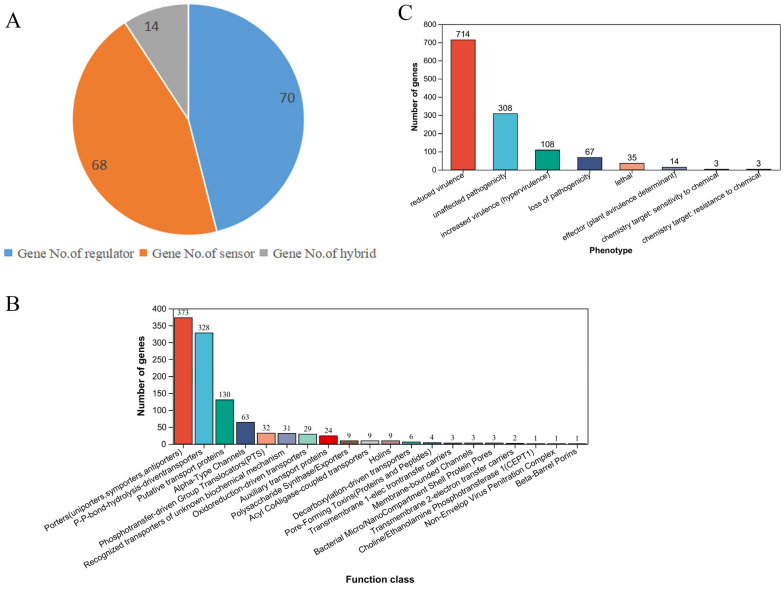
Intracellular regulation and functional protein analysis. (**A**) Two-component regulatory system analysis; (**B**) transporter protein analysis; (**C**) mutual analysis of pathogenic bacteria hosts.

The two-component regulation system usually transmits external signals to the response regulator by the sensor through phosphorylation. The response regulator can bind to DNA after phosphorylation, thereby promoting or inhibiting the transcription of specific genes [50]. The results of two-component regulation analysis show that a total of 70 regulatory factor genes, 68 sensor genes, and 14 mixed genes have been identified (Figure 6A). These regulatory genes mainly involve phosphate restriction, temperature, antibacterial peptide, environmental factors, malic acid, cationic antibacterial peptide, cell-wall-active antibiotics, salt coercion, low temperature, oxygen restriction, bacterial peptide, etc. (Figure S1). Based on the results of two-component regulation analysis, the strain shows remarkable environmental adaptability and can effectively respond to a variety of environmental coercion, including phosphate restriction, salt coercion, low temperature, and oxygen restriction. At the same time, the strain shows antibacterial tolerance and has the ability to form biofilm, which enhances its survivability. In addition, the existence of the sensor gene indicates that the strain can detect external chemical signals, thus regulating its metabolic activity [51]. In summary, the above characteristics lay an important foundation for an in-depth understanding of the ecological adaptation mechanism of the strain and its potential application.

#### 3.4.2. Analysis of Transporter Protein

Transporters are pivotal in biological processes such as nutrient absorption, metabolite transport, and environmental stress response. ABC transporters, in particular, are significant for pathogenicity, virulence expression [52], antibiotic synthesis [53], and probiotic properties [54] by controlling the intake of essential molecules and the secretion of toxigenic factors. The expression and activity of transporter proteins are regulated by internal and external signals, such as hormones, nutritional status, and temperature [55]. This study systematically analyzed the transport proteins of the strain. The results show that the 373 predicted transport proteins indicate that the strain has a diversified nutrient transport capacity, indicating that it can effectively obtain necessary nutrients under a variety of environmental conditions, thus supporting its survival and reproduction. In addition, the existence of 328 phosphate bond hydrolysis-driven transport proteins indicates that the strain can rely on hydrolysis for energy conversion and may rely on high-energy phosphoric acid compounds in its metabolic process, thus improving metabolic efficiency. The study also identified 130 hypothetical transport proteins, suggesting that the strain may not have been fully studied in some transport paths or there may be a potential new transport mechanism, which provides a broad exploration space for subsequent functional research.

At the same time, the 63 α-type channels and 29 redox-driven transport proteins found revealed the adaptability of the strain in responding to environmental changes, especially the survival potential under extreme conditions, which reflects its adaptation strategy to environmental pressure. In addition, the presence of 32 phosphorus transfer-driven population transport devices (PTS) and other transport proteins indicates that the strain has potential value in industrial applications, especially in the field of biotechnology such as fermentation and enzyme preparations (Figure 6B). In summary, the strain shows efficient nutrient acquisition ability and diversified transport mechanism, has good ecological adaptability, and shows important potential in industrial applications. These findings lay a solid foundation for subsequent biological research and application development and suggest its broad prospects in environmental and industrial applications.

#### 3.4.3. Analysis of the Interaction Between Pathogenic Microorganisms and Hosts

In the ecosystem, the interaction between pathogenic microorganisms and the host is a complex and dynamic process involving the pathogenic mechanism of microorganisms, the immune response of the host, and the influence of environmental and genetic factors [56]. This study systematically analyzes the virulence genetic characteristics of strains and reveals their high-quality characteristics in adaptability and pathogenic regulation. The discovery of 714 reduced virulence genes shows that strains can effectively regulate pathogenicity to adapt to different environments or hosts, which provides an important theoretical basis for the virulence regulation of pathogenic microorganisms. In total, 308 unaffected pathogenic genes maintain their persistent pathogenicity, suggesting that they still pose a potential threat under certain conditions. The existence of 108 virulence-enhancing genes indicates that the strain may have the ability to escape the host’s immune system, which is conducive to its reproduction and transmission in the host body, and provides a new perspective on the study of pathogenesis. An amount of 67 lost pathogenic bases provide a basis for evolutionary research, which may promote the strategy of non-thathogenic strains for biological prevention and control. Additionally, 35 lethal genes are associated with the pathogenesis of the host, laying the foundation for the study of the lethal mechanism of pathogenic bacteria. Finally, 14 effect factors indicate that the strain may regulate the host immune response through specific effectors, opening up application potential for the development of plant protectants [57] (Figure 6C). In summary, the virulence regulation flexibility and adaptability of the strain provide a broad space for basic research and the development of new prevention and control strategies, showing the application potential in agricultural disease management and public health.

### 3.5. Physiological Characteristics and Drug-Sensitivity Analysis of P. megaterium PH3

As the oldest form of life on earth, the diversity and complexity of microorganisms are an important research direction in biology. With the development of molecular biology technology, human cognition has expanded from taxonomy to the physiological characteristics and metabolic pathways, as well as their interaction with human health and the environment of microorganisms. In the field of medicine, drug-sensitivity experiments, hemolysis characteristics, metabolic ability, and bioamine research are crucial to understanding the infection mechanism of pathogenic microorganisms, optimizing clinical use and developing new antibacterial drugs. Through a series of classical microbiology experiments, this study comprehensively analyzes the physiological characteristics of strains and evaluates their response to commonly used antimicrobial drugs in order to provide a scientific basis for the diagnosis and treatment of infectious diseases.

#### 3.5.1. Research on Hemolytic Characteristics

**Figure 7:**
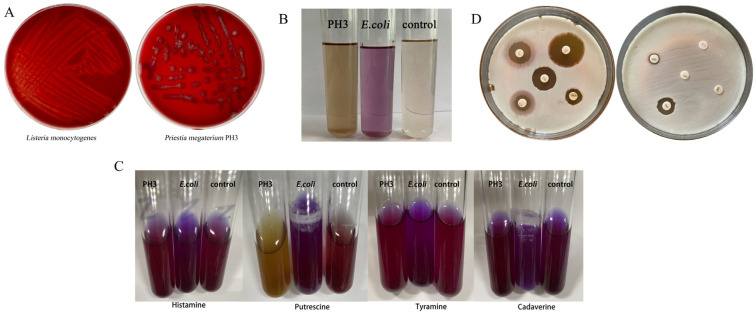
Safety evaluation. (**A**) Hemolysis experiment; (**B**) Indo matrix experimental tests; (**C**) biogenic amines experiment; (**D**) visualization of drug-sensitivity tests.

The hemolysis assay is an important indicator of the safety of a strain performed in vitro. Hemolysins cause the rupture of cell membranes, the lysis of cells, and direct damage to body tissues. If microorganisms carry hemolytic genes or hemolysins, they may cause sepsis or induce the development of other diseases [58]. The mechanism of this experiment mainly involves the destruction of red blood cells by hemolysins produced by bacteria. These hemolysins can be divided into α, β, and γ, which lead to partial dissolution, complete dissolution, or no hemolysis, respectively. They release hemoglobin by combining with the red blood cell membrane to form a hole or destroy the integrity of the membrane. In addition, some enzymes secreted by bacteria, such as phospholipase and hemolytic enzyme, can degrade the components of red blood cell membranes and improve the viability of bacteria in low-nutrition environments [59]. The hemolysis process can not only help bacteria escape the host’s immune response but also play an important role in the identification of bacteria and the diagnosis of infection. In this study, the hemolysis of the *P. megaterium* PH3 strain was inoculated into Colombian blood agar culture medium to observe its hemolysis. The results showed that in the positive control group, a transparent circle appeared around the colony, indicating the existence of β-hemolysis, while no transparent circle was observed around the colony of the *P. megaterium* PH3 strain, showing that it had no hemolytic ability (Figure 7A).

#### 3.5.2. Experimental Detection of Indole

The indole experiment is mainly used to detect whether bacteria can produce tryptophanase, which promotes the reductive deamination of tryptophan into indole. As an essential amino acid for the human body, tryptophan is crucial to many physiological functions, such as immune response and digestive processes. The abnormality of tryptophan metabolism may be related to a variety of health problems, including malignant tumors and liver failure [60]. Therefore, it is of great scientific significance to explore the characteristics of specific bacteria in tryptophan metabolism to understand the mechanism of related diseases [61]. In this experiment, *E. coli* was taken as the positive control group, and the uninoculated sample was taken as the negative control group. The results showed that the culture solution of the positive control group reacted with the color developer because it could produce indole, presenting rose red, while the negative control group remained light yellow (Figure 7B). The culture solution of the experimental group *P. megaterium* PH3 is yellow, indicating that the *P. megaterium* PH3 strain does not have tryptophanase activity, so it will not have a significant impact on the host’s immune function and digestion process.

#### 3.5.3. Detection of the Ability to Produce Bioamines

Bioamine is a class of low-molecular-mass organic compounds that can react with nitrite to produce the carcinogenic substance nitroamine. Excessive doses will poison the human body [62]. Research shows that if microorganisms metabolize amino acid decarboxylase, they can decarboxylase amino acids to form biological amines, which has adverse effects [63]. In microbiology research, the ability to produce bioamines has received more and more attention. Bioamines are organic compounds produced by amino acid decarboxylation, which are widely found in nature, especially in fermented foods and some rotten foods [64]. Some microorganisms can synthesize bioamines during their metabolism, which not only affects the flavor and quality of food but also may have an important impact on food safety and human health [65]. This study aims to determine the ability of specific strains to produce bioamines to evaluate their applicability and safety in food processing and fermentation. The experimental results showed that compared with the uninoculated negative control group, the positive control group inoculated with *E. coli* showed a purple reaction in the process of bioamine determination, while the experimental group inoculated with *P. megaterium* PH3 showed yellow or no obvious color change (Figure 7C). From this, it can be inferred that the *P. megaterium* PH3 strain does not have amino acid decarboxylase activity in its metabolic process, so it does not produce biological amine-harmful substances. Through in-depth research on the ability of the strain to produce bioamines, the results obtained lay the foundation for the further research and practical application of the strain *P. megaterium* PH3 in related fields.

#### 3.5.4. Research on Drug Sensitivity

A drug-sensitivity test is a key step in microbiology research, which aims to evaluate the sensitivity of specific bacteria to a series of antibiotics. In this study, we systematically evaluated the drug sensitivity of the bacterial strain *P. megaterium* PH3 and analyzed the sensitivity of commonly used antibiotics using the K–B disk diffusion method [66]. The results showed that the *P. megaterium* PH3 strain showed high sensitivity to chloramphenicol, indicating that the use of chloramphenicol in clinical treatment of related infections has a good effect. After that, ciprofloxacin and cephalosporin also showed good sensitivity, indicating that these antibiotics are also suitable for the treatment of *P. megaterium* PH3 strains. However, drug resistance is also important information that cannot be ignored in our results. The resistance of *P. megaterium* PH3 to penicillin, ampicillin, and lincomycin suggests the potential threat of the current bacteria to these commonly used antibiotics, which not only affects the treatment effect but also poses challenges to clinicians’ treatment choices (Figure 7D; Table 5).

### 3.6. Toxicological Analysis of P. megaterium PH3

As part of biological agents, the safety assessment of bacteria is particularly important because they may directly or indirectly affect human health. Mouse models are often used as model organisms to evaluate bacterial safety because of their physiological and genetic similarities with humans [9]. Through mouse experiments, we can evaluate the possible effects of bacteria in the human body so as to predict its potential impact on the human body. This study aims to observe the effect of strains on mice and evaluate whether they are toxigenic, pathogenic, or cause other adverse reactions in organisms.

#### 3.6.1. The Effect of *P. megaterium* PH3 on the Basic Indicators of Mice

**Figure 8:**
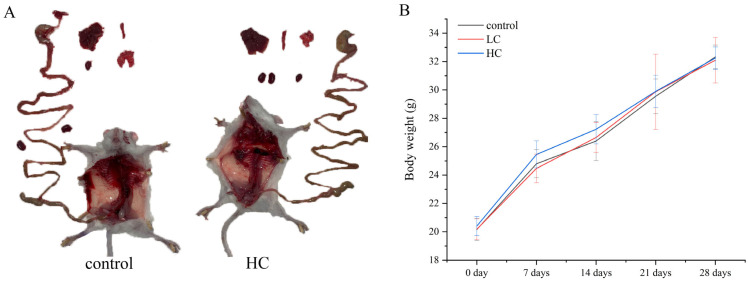
Results of *P. megaterium* PH3 on basal indices in mice. (**A**) Organ tissues of mice; (**B**) body weight of mice (low-dose group (1.5 × 10^5^ CFU/mL, LC), and high-dose group (1.5 × 10^10^ CFU/mL, HC)).

In this study, we observed the general characteristics of mice in detail, and the potential impact of bacteria on the neurological function of mice can be reflected through behavioral performance. The results showed that the mice in the experimental group showed good condition in terms of behavior, mental state, and physiological indicators. These results were compared and analyzed with the blank group. We found that there was no abnormality in the behavior of the mice. The mental state was positive and the fur color was bright, which are important indicators of health status [67]. In addition, normal diet and drinking water, as well as normal excretion (such as granular feces), further confirm the overall health of the mice. It is worth noting that no significant abnormality was found in the naked eye observation after autopsy, and the results showed that *P. megaterium* PH3 did not cause observable damage to the organs and tissues of mice (Figure 8A).

In addition, weight, as an intuitive indicator reflecting the health status of animals, can reveal the impact of bacteria on the growth and development of mice. Through the measurement of the weight of mice, the results showed that there was no significant difference compared with the blank control group, which further supported the conclusion that the strain *P. megaterium* PH3 had no significant effect on the growth and development of mice (Figure 8B). These results show that the strain *P. megaterium* PH3 has no significant effect on the growth and development of mice under experimental conditions.

#### 3.6.2. The Effect of *P. megaterium* PH3 on the Organ Index of Mice

**Figure 9:**
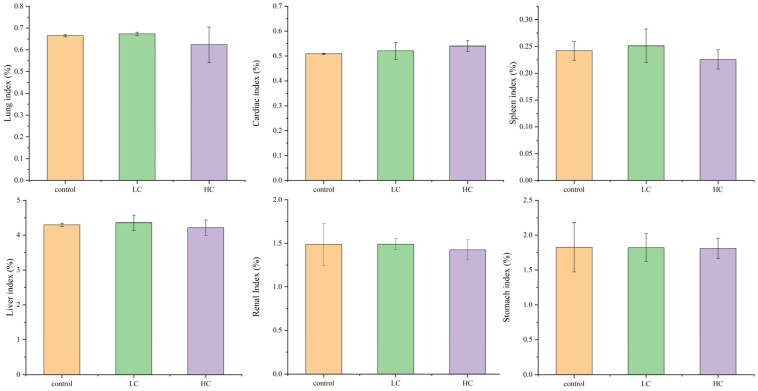
Mice organ index analysis (LC stands for low-dose group (1.5 × 10^5^ CFU/mL) and HC stands for high-dose group (1.5 × 10^10^ CFU/mL)).

The organ indicators of mice can reveal their health status and metabolic status. The organ index provides quantitative data on the effect of bacteria on the organ function of mice by comparing the ratio of organ weight to body weight. In addition, researchers can also evaluate the metabolic status of mice by analyzing organ indicators. At the same time, observing the changes in the size of the organs is helpful to assess the progress of the disease. Changes in the spleen provide important information about the immune status of mice [68]. In addition, the analysis of the specific gravity of organs can also be used to observe the health of the growth and development of mice. In summary, organ indicators have important application value in mouse health assessment and disease research. The results showed that the strain *P. megaterium* PH3 had no significant effect on the liver, kidney, heart, spleen, stomach, lungs, and other organs of mice (Figure 9). The above results show that the strain *P. megaterium* PH3 has no significant effect on the physiological function and health status of mice. This discovery supports the reliability of the strain *P. megaterium* PH3 in terms of safety, implying that it may have good safety characteristics in further research or application.

#### 3.6.3. The Effect of *P. megaterium* PH3 on Organ Lesions in Mice

**Figure 10:**
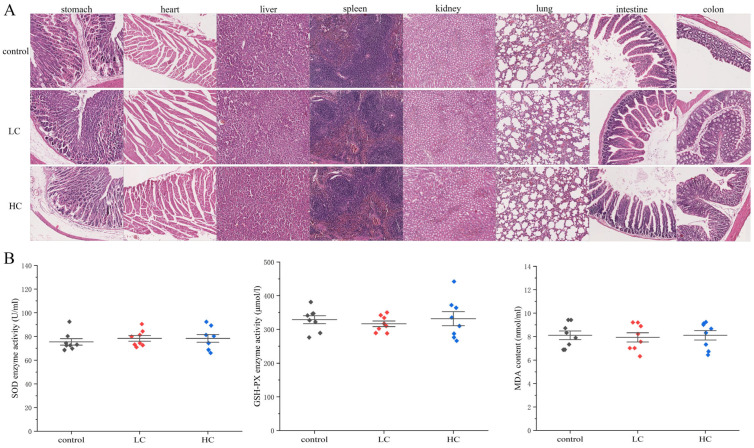
Pathological analysis of *P. megaterium* PH3 on mice. (**A**) Organ slices; (**B**) oxidative stress analysis in serum (LC stands for low-dose group (1.5 × 10^5^ CFU/mL) and HC stands for high-dose group (1.5 × 10^10^ CFU/mL)).

By detecting the pathological changes of mouse organs, the potential toxigenic effects of the strain *P. megaterium* PH3 on the organs are evaluated, so as to provide a reliable basis for its safety [69]. In order to further explore the safety of *P. megaterium* PH3, pathological sections were carried out on organs such as the liver, kidney, heart, spleen, stomach, small intestine, colon, and lung, respectively. In the study, we conducted a systematic pathological section observation of the main organs to identify whether there were any abnormal changes. The results showed that no significant lesions were found in tissue sections of various organs in mice fed with *P. megaterium* PH3 after gastric lavage (Figure 10A). The above results show that the strain *P. megaterium* PH3 did not cause obvious tissue damage or pathological changes to the main organs of mice under experimental conditions, revealing that it has certain good characteristics in terms of safety.

#### 3.6.4. The Effect of *P. megaterium* PH3 on the Oxidative Stress Level of Mice

Changes in oxidative stress markers often occur before pathological changes, so they can be used as early warning indicators to help detect potential risks in time [70]. The oxidative stress factor in serum can reflect the physiological state of the whole body, which is crucial for evaluating the systemic safety of the strain. The horizontal changes of these factors can indicate the body’s stress response to external stimuli and the functional state of the antioxidant defense system [71]. By determining the activity of key oxidative stressors in mouse serum samples, the level of oxidative stress caused by bacteria can be evaluated, which is crucial for understanding the potential toxicity of bacteria. The results showed that *P. megaterium* PH3 did not have a significant effect on the enzymatic activity of SOD and GSH in mouse serum. It shows that *P. megaterium* PH3 did not cause excessive reactive oxygen production in the metabolic process of mice or did not put pressure on the body’s antioxidant defense mechanism. In addition, the no significant change in MDA content further confirms that *P. megaterium* PH3 has no negative impact on the serum lipid peroxidation level of mice, which is consistent with the stable results of antioxidant enzyme activity. By combining these results, we can conclude that the strain *P. megaterium* PH3 did not cause obvious oxidative stress damage to the mouse body under the experimental conditions of this study (Figure 10B).

## 4. Conclusions

In this study, a comprehensive genome and functional analysis of *P. megaterium* PH3 was carried out through whole-genome sequencing, and it was confirmed that its genome was composed of one chromosome and seven plasmids. A total of 6132 coding genes were identified, with a GC content of 37.62%. Functional analysis shows that the important bases related to carbohydrate metabolism, secondary metabolite synthesis, and pathogenesis provide a theoretical basis for the biosynthesis of resveratrol. In addition, the analysis of the two-component signal transduction system shows that the strain shows significant adaptability under environmental pressure. The interaction analysis of host plants revealed 714 weakening genes that regulate the host’s immune response, further supporting their potential applications in the development of plant protectants. Safety evaluation results show that *P. megaterium* PH3 is non-hemolytic, does not produce amino acid decarboxylase, has no significant resistance to common antibiotics, and shows good physiological safety in mouse models. Therefore, *P. megaterium* PH3 deserves further research and development with its excellent security and wide application prospects. Especially in the strategy of optimizing resveratrol production and improving plant disease resistance, this strain has laid important basic data for future applications.

## Figures and Tables

**Table 1:** Genome assembly results.

Parameter	Value
Total Bases (bp)	4,318,106,144
Largest (bp)	317,278
Chromosome No.	1
Plasmid No.	7
Genome Size (bp)	5,960,365
G + C (%)	37.62
Reads N50 (bp)	11,391

**Table 2:** Genome prediction results.

Type	Parameter	Value
Gene	Gene Total Len (bp)	4,877,301
	GC Content in Gene Region (%)	38.59
	Gene/Genome (%)	81.83
	Gene Average Len (bp)	795.39
tRNAs	tRNAs	140
rRNAs	16S rRNA	15
	23S rRNA	15
	5S rRNA	17
sRNA	sRNA No	115
	Total Len (bp)	17,949
	In Genome (%)	0.3011
Methylation modification	M4C; modified_base; m4C; m6A	4

**Table 3:** Secondary metabolite synthesis gene cluster results.

Cluster ID	Location	Type	Gene No.	MIBiG Accession
Cluster1	PlasmidB	lanthipeptide-class-i	25	-
Cluster2	Chromosome	terpene	20	carotenoid
Cluster3	Chromosome	phosphonate	35	-
Cluster4	Chromosome	T3PKS	37	-
Cluster5	Chromosome	terpene	20	-
Cluster6	Chromosome	terpene	22	surfactin
Cluster7	Chromosome	siderophore	15	-

Note: MIBiG represents minimum information about a biosynthetic gene cluster.

**Table 4:** Results of resistance gene prediction.

Parameters		Gene Information
Description Info	Gene Name	lsa
	Location	Plasmid E: complement (26,583–28,061)
	Description	Lsa family ABC-F type ribosomal protection protein

**Table 5:** Results of drug-sensitivity tests.

Name	Drug Content (μg)	Diameter of Judgement Criteria (mm)			Diameter (mm)	Grade
		R	I	S		
Penicillin (PEN)	10	≤28	-	≥29	0	R
Chloramphenicol (CLM)	10	≤16	18~24	≥25	20.67 ± 0.31	I
Erythromycin (ERM)	30	≤13	14~20	≥21	4.07 ± 0.01	R
Tetracycline (TET)	30	≤14	15~16	≥17	7.47 ± 0.13	R
Gentamicin (GEN)	15	≤13	14~22	≥23	6.67 ± 0.12	R
Lincomycin (LIM)	5	≤15	16~20	≥17	0	R
Ciprofloxacin (CFX)	10	≤12	13~14	≥15	10.67 ± 0.12	R
Ceftriaxone (CTR)	23.75	≤10	11~15	≥16	12.67 ± 0.12	I
Ampicillin (AMP)	30	≤12	13~17	≥18	0	R
Paediatric Compound Sulfamethoxazole Tablets (T/S)	2	≤13	14~17	≥18	4.67 ± 0.12	R

Note: R: resistance; I: intermediary; S: sensitivity.

## Data Availability

The original contributions presented in the study are included in the article and Supplementary Materials, further inquiries can be directed to the corresponding author.

## Supplementary Materials

The following supporting information can be downloaded at: https://www.mdpi.com/article/10.3390/foods13223555/s1, Figure S1: Diagram of two-component system regulation analysis.
